# Effectiveness of sigmoidoscopy for assessing ulcerative colitis disease activity and therapeutic response

**DOI:** 10.1097/MD.0000000000015748

**Published:** 2019-05-24

**Authors:** Wei-Chen Lin, Chen-Wang Chang, Ming-Jen Chen, Tzu-Chi Hsu, Horng-Yuan Wang

**Affiliations:** a Division of Gastroenterology, Department of Internal Medicine, Mackay Memorial Hospital and Mackay Medicine, Nursing and Management College; b Division of Colon and Rectal Surgery, Mackay Memorial Hospital, Taipei, Taiwan.

**Keywords:** disease activity, sigmoidoscopy, ulcerative colitis

## Abstract

Ulcerative colitis (UC) typically begins in the rectum and progresses proximally in a contiguous fashion without skip lesions. Post-treatment inflammation distribution can change over time. Colonoscopy is unpleasant for the patient and clinical trials often use sigmoidoscopy for evaluation of disease severity. The aim of this study is to evaluate whether sigmoidoscopy is adequate to assess disease activity and therapeutic response as colonoscopy.

We retrospectively reviewed patients who underwent colonoscopy for the initial diagnosis and follow-up by evaluating their mucosal inflammation in our hospital from January 2012 and December 2017.

A total of 69 patients were analyzed. During follow up, the inflamed segment changed post-treatment in 62% (43/69). Extensive UC was common in the changed disease extent group (*P* < .01). Patients treated with oral mesalazine had a higher rate of changed disease extent (*P* < .01). The sigmoid segment was the most commonly involved segment, and the rectum was the severely inflamed segment during initial diagnosis and follow-up. According to Mayo endoscopic subscore (MES) in the most severely inflamed colonic and rectosigmoid segment, there were high degrees of correlation in the initial UC diagnosis (*r* = .90, *P* < .01) and follow-up (*r* = .74, *P* < .01).

Our findings suggest that sigmoidoscopy is effective as colonoscopy for detecting disease activity and evaluating therapeutic response in UC patients during follow-up.

## 1 Introduction

Ulcerative colitis (UC) is a chronic inflammatory bowel disorder characterized by exacerbations and remissions. Colonoscopy with intubation of the terminal ileum is the cornerstone for the initial diagnosis and evaluation of UC disease extent and activity.^[1]^ With the development of medical therapies for UC, achieving mucosal healing has become an important therapeutic objective in clinical remission and reducing surgeries and hospitalizations.^[1,2]^ The first timing to evaluate the response to biologics is around 2 to 3 months of induction therapy following initial infusions.^[2]^ The secondary timing of endoscopy is 52 weeks in a clinical trial, in patients whose diseases are refractory to treatment and require therapeutic changes, or for cancer surveillance.^[1,2]^ In Taiwan, due to budget limitations, the National Health Insurance only allows for a limited period of biologics use in UC patients and requires endoscopic follow-up 2 months after the induction of therapy and then every 4 months of maintenance therapy to evaluate disease activity.^[3]^ More frequent surveillance endoscopy is predictable for the altered therapeutic paradigm in the near future.

An adequate range of colonic observations for the precise evaluation of inflammation is controversial, and some patients may have more severe inflammation proximal to the sigmoid colon.^[4,5]^ Many guidelines and clinical trials have allowed for the endoscopic assessment of UC using sigmoidoscopy rather than colonoscopy without providing clear justification.^[5,6]^ The most severe UC activity is usually seen in the distal colon; thus, endoscopic assessment of the rectosigmoid segment is standard.^[6]^ Few studies have compared the efficacy of sigmoidoscopy with that of colonoscopy for assessing disease activity.^[4,7]^ Endoscopy is unpleasant for patients; therefore, 1 study investigated whether non-invasive disease activity index (partial Mayo score) could be used to predict disease activity and avoid endoscopy.^[8]^ Several recent studies discussed faecal calprotectin as a surrogate and reliable marker of endoscopic remission.^[9,10]^ On the other hand, the utility of endoscopy for assessing mild to moderate UC remains debatable, and most patients can be managed according to clinical symptoms alone.

The primary outcome of this study was the ability to evaluate the influence of therapeutic approaches on each colonic site and identify the factors associated with changes in disease extent. The secondary outcome was to establish whether sigmoidoscopy was equivalent to colonoscopy for assessing UC disease activity and therapeutic response.

## 2 Materials and methods

### 2.1 Patient selection

UC patients who underwent a colonoscopy at least twice at Mackay Memorial Hospital, Taipei Medical Center, between January 2012 and December 2017 were retrospectively analyzed. The diagnosis of UC was based on a clinical evaluation of the patient's medical history, clinical findings, and typical endoscopic and histological findings. The first timing of colonoscopy was for initial diagnosis. The second timing was at the symptom onset or remission after medical therapy. Patients who had no good-quality whole colonic segment, had undergone colectomy, had a history of recent cytomegalovirus or *Clostridium difficile* infection, or had recently used nonsteroidal anti-inflammatory drugs were excluded from the study. Medical charts provided clinical parameters including demographic data (age, sex, family history, smoking habit) and disease status (disease activity, location, and duration). Medical history included mesalazine (oral, enema or suppository), steroids, immunosuppressants, and biologics use within the previous 4 weeks. This study was approved by the Institutional Review Board of Mackay Memorial Hospital (reference number: 15MMHIS044), which waived the requirement for informed consent because of its retrospective design. Patient information was anonymized and de-identified prior to the analysis.

### 2.2 Data collection and variable definitions

Endoscopic findings were evaluated by examining recorded colonoscopy images. Each location (rectum, sigmoid colon, descending colon, transverse colon, and ascending colon) was examined in each patient. The colonic site with maximum inflammation was determined by Mayo endoscopic subscore (MES) defined as follows: normal (0 points); erythema, decreased vascular pattern, mild friability (1 point); absent vascular pattern, friability, erosions (2 points); and spontaneous bleeding or ulceration (3 points).^[11]^ Two experienced endoscopists examined the images independently and determined the scores without adjudication. The MES of each colonic segment was defined by the severely inflamed location and recorded separately. Disease activity according to colonoscopy and sigmoidoscopy scores was defined as the MES in the most severely inflamed segment. The non-inflamed segment was defined as a MES of ≤1. Disease location change was defined as the initial inflamed or non-inflamed area transferred to a non-inflamed or inflamed segment after medical therapy.

### 2.3 Statistical analysis

Descriptive statistics for continuous variables are reported as mean ± standard deviation (SD). Categorical variables are described using frequency distributions and reported as n (%). *P* values were based on a *t* test for continuous variables and the chi-square test or Fisher exact test for categorical variables. The highest MES of each colonic and rectosigmoid segments were correlated using Spearman coefficient of correlation. The statistical analysis was performed using the STATA statistical package (version 13.0; Stata, College Station, TX). All *P* values are 2-sided and those of *P* < .05 were considered statistically significant.

## 3 Results

### 3.1 Demographic features

There were 137 UC patients under treatment in our hospital; of them, 69 who had undergone colonoscopy for the initial diagnosis and follow-up were recruited. The median colonoscopy follow-up duration was 3 years (range, 0.5–5 years). Eleven (16%) patients achieved endoscopic remission (MES ≤ 1) on the follow-up endoscopy. The clinical characteristics of these patients are provided in Table 1. Among them, 58% were male with a median age at diagnosis of 41 years. With regard to disease location at the initial diagnosis, extensive UC was the most common (46%), followed by proctitis (33%) and left-sided colitis (20%). The inflamed locations in 43 (62%) patients changed after medical treatment. Among the findings, the most common was inflammatory changes of the proximal segment (38%), followed by segmental skip (14%) and distal segment (10%).

**Table 1:**
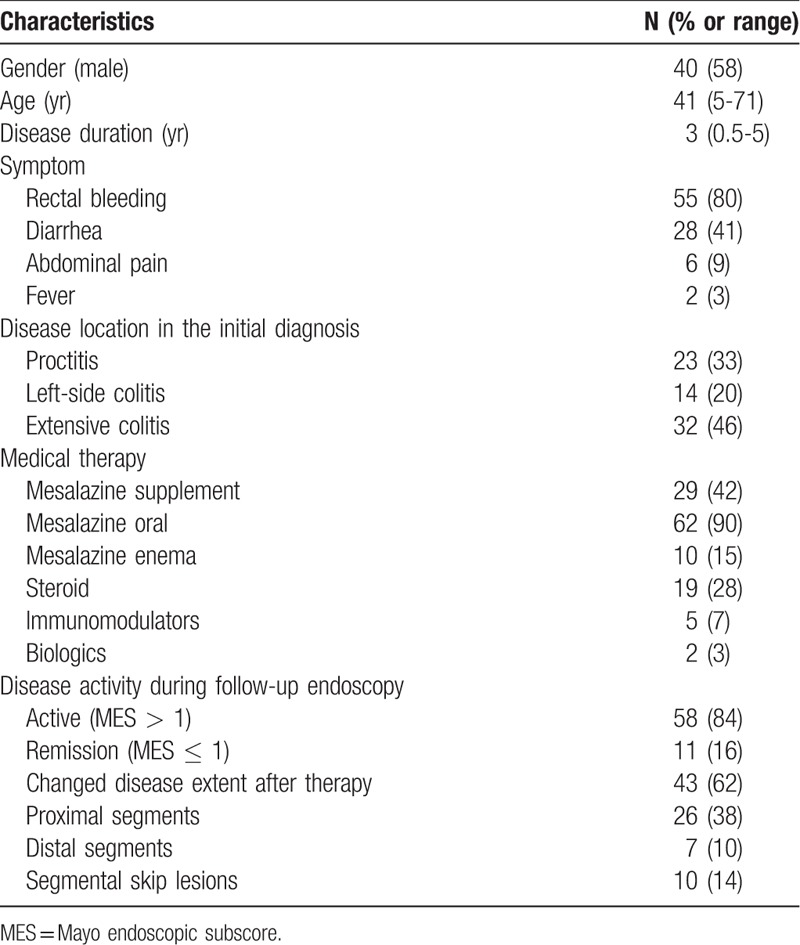
Characteristics of 69 ulcerative colitis patients who underwent colonoscopy for the initial diagnosis and follow-up.

### 3.2 Comparison of patients with and without changed disease extent

The demographic and characteristics of the UC patients with and without changed disease extent are summarized in Table 2. There were no significant intergroup differences in age, sex, family history, smoking, or disease duration. Extensive UC was common in the changed disease extent group (23% vs 60%, *P* < .01), while proctitis was common in the non-changed disease extent group (62% vs 16%, *P* < .01). After medical treatment, oral mesalazine was more commonly prescribed in the changed disease extent group (73% vs 100%, *P* < .01). Rectal therapy, steroid, immunosuppressants, and biologics use did not differ significantly between groups.

**Table 2:**
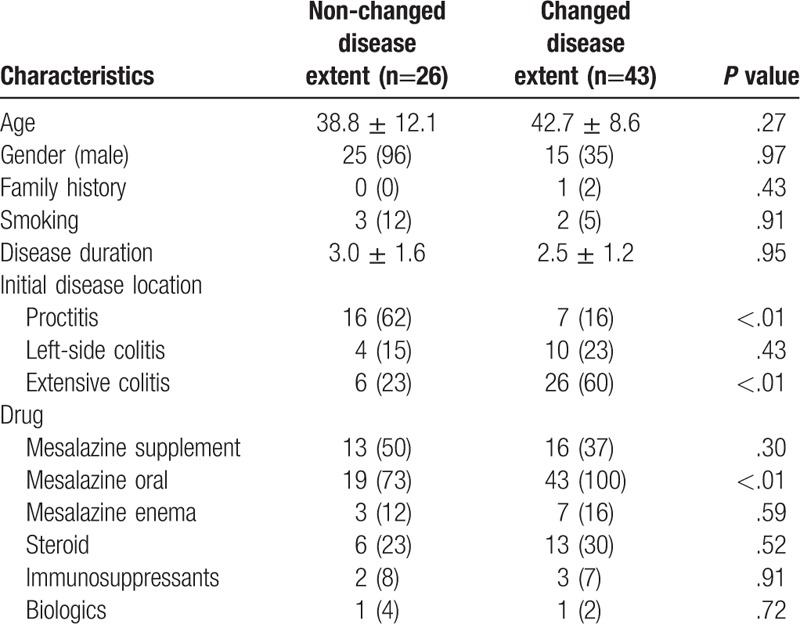
Comparisons of characteristics between ulcerative colitis patients with and without changed disease extent.

### 3.3 Correlation between sigmoidoscopy and colonoscopy

**Figure 1:**
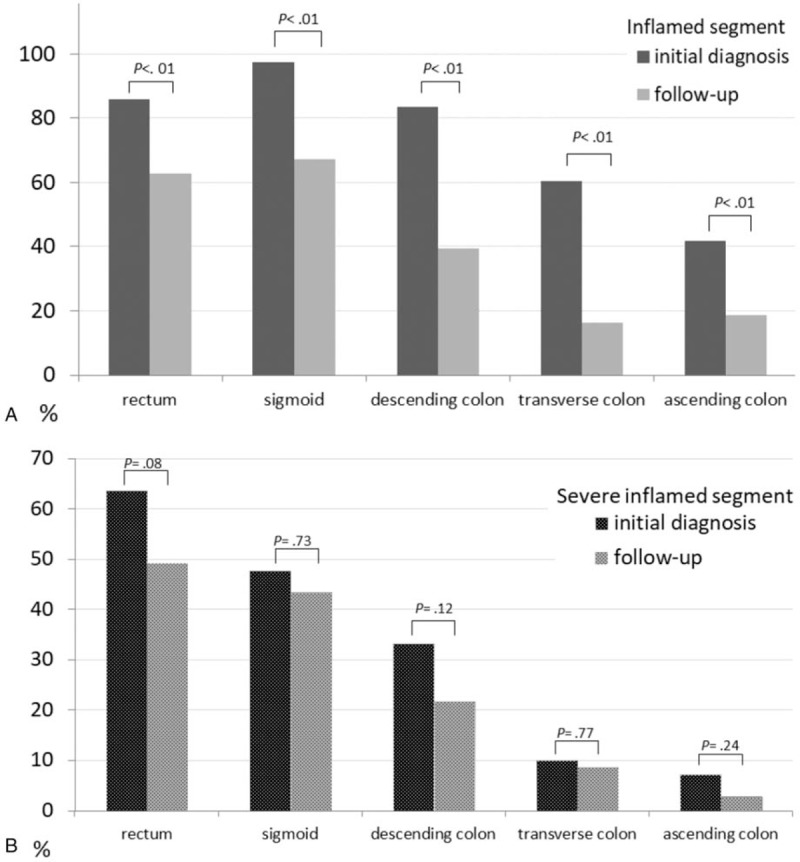
Distribution of the inflamed (A) and severely inflamed locations (B) in each colonic location of 69 patients with ulcerative colitis during initial diagnosis and follow-up endoscopy.

**Figure 2:**
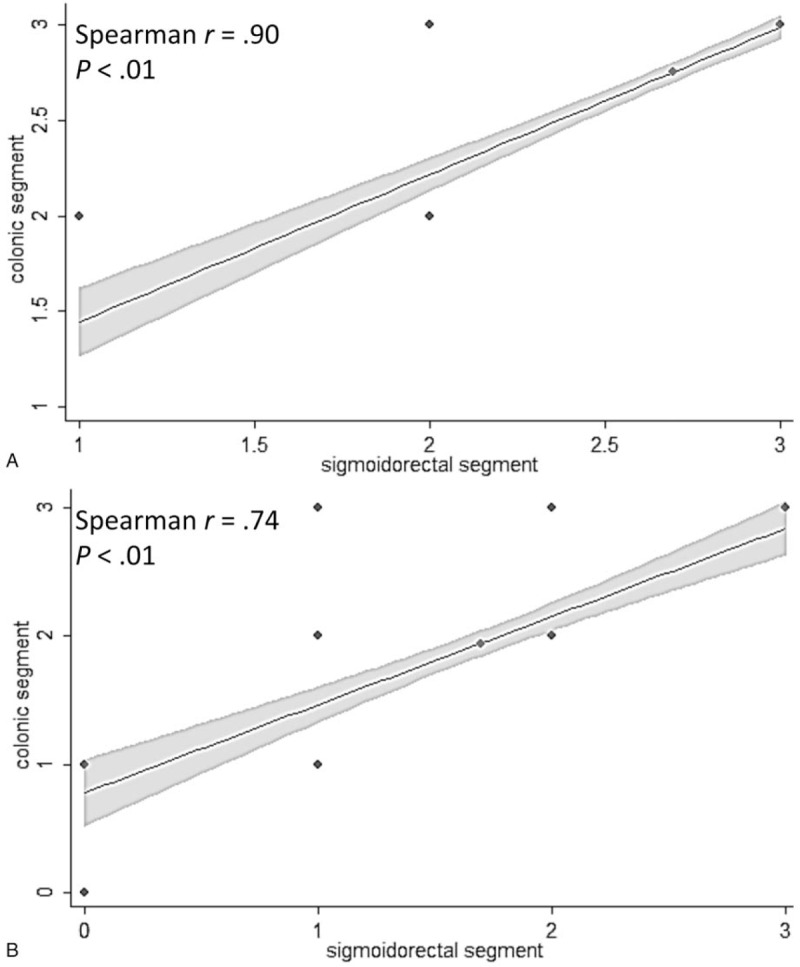
Scatterplot showing correlation between Mayo endoscopic subscores of colonic and sigmoidorectal segments at A. initial diagnosis; and B. follow-up.

The sigmoid was the most common inflamed colonic segment at the time of the initial UC diagnosis (99%, 68/69 patients), followed by the rectum (Fig. 1A). The inflamed location was significantly decreased in each colonic segment after therapy. The sigmoid segment was the most commonly inflamed segment after treatment, as it occurred in 80% (53/69) of the patients. As for the severely inflamed segments, the rectum and descending colon decreased much after therapy but without statistical significance (*P* = .08 and *P* = .12; Fig. 1B). The rectum was the most severely inflamed segment at the time of the initial diagnosis and medical therapy. The mean MES in each colonic and rectosigmoid segment at the time of the initial diagnosis were 2.8 and 2.7 points versus 1.9 and 1.7 points during UC follow-up. Regarding the correlation coefficients of MES between each colonic and rectosigmoid segment, there was statistically significant positive correlations between the initial UC diagnosis (*r* = .90, *P* < .01) and the UC follow-up (*r* = .74, *P* < .01) (Fig. 2).

## 4 Discussion

An important element of the present study is that disease activity was determined in each of the 5 colonic segments to evaluate the extent of the affected locations during initial diagnosis and follow-up. We found that the most severely inflamed segment was the rectum, and the sigmoid colon was the most commonly inflamed segment after therapy. Our results also showed that MES on sigmoidoscopy was significantly correlated with that on colonoscopy during follow-up, revealing the sigmoidoscopy was effective for the assessment of disease activity and therapeutic response of UC in the clinical practice.

UC is a state of chronic continuous inflammation that extends proximally from the rectum, where it is the most severe. The UC endoscopic index is determined using the most severely affected segment in the colorectum.^[1,6]^ Although endoscopy is recognized as the most reliable method for evaluating disease activity, that examination is relatively invasive and sometimes painful. In special groups such as the elderly, colonoscopy carries a greater risk of complications.^[12]^ Furthermore, colonoscopy can increase UC symptoms, most commonly 1 week immediately after the procedure.^[13]^ Colonic preparation with sodium phosphate has showed to cause left-sided colonic mucosal ulcerations.^[14]^ Glutaraldehyde is the standard chemical used to disinfect the equipment, and residual glutaraldehyde on endoscopes has been found to be associated with colitis.^[15]^ However, international consensus recommends that endoscopic, clinical, and histological scoring systems are the key components used to determine clinical response and/or remission to medical intervention.^[6]^ Sigmoidoscopy rather than colonoscopy might be useful in clinical practice to assess disease activity with the growing use of endoscopy.

UC typically begins in the rectum and progresses proximally in a contiguous fashion. In the initial diagnosis of UC, one study showed that around 19.2% of patients had atypical distributions.^[16]^ Rectal sparing, the most common atypical presentation, was seen in 9% of our patient population. A previous study showed that rectal sparing may be due to relatively minor inflammatory findings on an endoscopic examination, but no patients displayed complete absence of inflammation in the pathologic features of the colectomy specimens.^[17]^ Segmental skip lesions were more frequently observed in the proximal segments of the colon (transverse and ascending colon).^[16]^ Proximal extension of UC was reported in 30% of patients on follow-up endoscopy.^[18]^ A more severe disease status at initial diagnosis was associated with disease extension.^[19]^ Therefore, disease extent would change over time and require monitoring.

In this study, there was a high degree of correlation between sigmoidoscopy and colonoscopy findings in the follow-up as at the time of the initial diagnosis. One study showed that sigmoidoscopy was adequate in terms of the strict definition of mucosal healing (MES = 0) but that colonoscopy was better in cases with an MES ≤1.^[7]^ Another endoscopic study of 545 UC patients showed that 40% patients had an inflamed mucosa in the descending colon without rectosigmoid segment involvement after therapy.^[4]^ They concluded that colonoscopy was necessary if the sigmoidoscopy findings did not correlate with the patient's symptoms.^[4]^ Noteworthy, the higher disease remission rate (55%) in that study was quite different from the lower remission disease status (16%) during follow-up endoscopy in our study. Therefore, the use of sigmoidoscopy to assess treatment-responsive active UC was suitable.

After longstanding treatment, the extent and the distribution of inflammation in UC may vary significantly, including a reversion to normal mucosa. One study revealed that endoscopic patchy inflammation and rectal sparing was not uncommon; rather, it occurred in 59% of patients.^[20]^ The cecum and periappendix is the common patchiness location.^[20]^ The patchy disease occurred in 30% of UC patients by endoscopy, but a poor correlation was found between the endoscopic and pathologic features in colectomy specimens.^[17]^ Therefore, this study revealed no correlation between the use of any specific anti-inflammatory medications and the presence of patchiness under pathologic features.^[17]^ The presence of isolated peri-appendiceal lesions was not a risk factor for the escalation of UC therapy and was not correlated with proximal disease extension.^[21]^ Therefore, colonoscopy is not strongly indicated in such situations.

Oral therapy can induce segmental healing and patchiness of inflammatory activity.^[20]^ The most common drug related to patchy inflammation was oral mesalazine, followed by oral steroids.^[20]^ This result was compatible with our study revealing the common drug of changed disease extent was oral mesalazine. Rectal therapy is widely recognized to cause rectal sparing.^[22]^ In a study of 39 patients with treated UC, 13% of patients had endoscopic and 15% had histopathologic findings of rectal sparing.^[23]^ In the era of biologics (such as etrolizumab), most patients (95%) still had greater disease severity in the distal colon after therapy, and this study concluded that sigmoidoscopy was adequate for assessing disease activity.^[7]^

### 4.1 Limitations

There are several potential limitations to this study. First, the small sample size and retrospective design might have led to patient selection bias. Patients with severe active disease in the initial diagnosis were excluded due to colonoscopy not being performed to prevent toxic megacolon. Several follow-up endoscopies were performed during the recurrence of symptoms, and patients with clinical remission might not have all segmental images. Fewer patients received potent drugs, which caused the lower endoscopic remission rate in this study. Second, with regard to the disease extent response to medical therapy, most patients received combination therapy, which might make a solid conclusion about the different impacts of each therapy difficult to reach. Finally, we did not perform histological examinations of the colonic biopsy samples, which might have led to underestimating of the disease activity due to endoscopic findings not always being consistent with histological activity.^[17]^ Further investigations based on histological examination findings obtained from all colonic segments should be performed to validate our findings.

## 5 Conclusions

With the advanced medical therapy of UC, the more frequency use of objective tools such as endoscopy to evaluate clinical remission and mucosal healing is urgently needed. Our findings suggest that sigmoidoscopy is equally as effective as colonoscopy for assessing UC disease activity and treatment response.

## Acknowledgments

The authors would like to thank all gastroenterology faculty of MacKay Memorial Hospital for excellent clinical assistance and care.

## Author contributions

**Conceptualization:** Wei-Chen Lin.

**Data curation:** Chen-Wang Chang.

**Investigation:** Ming-Jen Chen.

**Methodology:** Wei-Chen Lin, Chen-Wang Chang.

**Resources:** Tzu-Chi Hsu.

**Supervision:** Ming-Jen Chen, Tzu-Chi Hsu, Horng-Yuan Wang.

**Validation:** Chen-Wang Chang, Ming-Jen Chen.

**Visualization:** Horng-Yuan Wang.

**Writing – original draft:** Wei-Chen Lin.

**Writing – review & editing:** Horng-Yuan Wang.

Wei-Chen Lin orcid: 0000-0002-8142-538X.
